# DNA Methylation Profiling of Human Prefrontal Cortex Neurons in Heroin Users Shows Significant Difference between Genomic Contexts of Hyper- and Hypomethylation and a Younger Epigenetic Age

**DOI:** 10.3390/genes8060152

**Published:** 2017-05-30

**Authors:** Alexey Kozlenkov, Andrew E. Jaffe, Alisa Timashpolsky, Pasha Apontes, Sergei Rudchenko, Mihaela Barbu, William Byne, Yasmin L. Hurd, Steve Horvath, Stella Dracheva

**Affiliations:** 1 James J. Peters VA Medical Center, Bronx, NY 10468, USA; alexey.kozlenkov@mssm.edu (A.K.); alisa.timashpolsky@gmail.com (A.T.); p.apontes@gmail.com (P.A.); william.byne@mssm.edu (W.B.); 2 The Friedman Brain Institute and Department of Psychiatry, Icahn School of Medicine at Mount Sinai, New York, NY 10029, USA; yasmin.hurd@mssm.edu; 3 Lieber Institute for Brain Development, Johns Hopkins Medical Campus, Baltimore, MD 21205, USA; andrew.jaffe@libd.org; 4 Department of Biostatistics and Department of Mental Health, Johns Hopkins Bloomberg School of Public Health, Baltimore, MD 21205, USA; 5 Center for Computational Biology, Johns Hopkins University, Baltimore, MD, 21205, USA; 6 Hospital for Special Surgery, New York, NY 10021, USA; RudchenkoS@hss.edu (S.R.); mihaela72000@yahoo.com (M.B.); 7 Department of Human Genetics, David Geffen School of Medicine, University of California Los Angeles, Los Angeles, CA 90095, USA; shorvath@mednet.ucla.edu; 8 Department of Biostatistics, School of Public Health, University of California Los Angeles, Los Angeles, CA 90095, USA

**Keywords:** DNA methylation, drug addiction, heroin, suicide, brain, neurons, human

## Abstract

We employed Illumina 450 K Infinium microarrays to profile DNA methylation (DNAm) in neuronal nuclei separated by fluorescence-activated sorting from the postmortem orbitofrontal cortex (OFC) of heroin users who died from heroin overdose (*N* = 37), suicide completers (*N* = 22) with no evidence of heroin use and from control subjects who did not abuse illicit drugs and died of non-suicide causes (*N* = 28). We identified 1298 differentially methylated CpG sites (DMSs) between heroin users and controls, and 454 DMSs between suicide completers and controls (*p* < 0.001). DMSs and corresponding genes (DMGs) in heroin users showed significant differences in the preferential context of hyper and hypo DM. HyperDMSs were enriched in gene bodies and exons but depleted in promoters, whereas hypoDMSs were enriched in promoters and enhancers. In addition, hyperDMGs showed preference for genes expressed specifically by glutamatergic as opposed to GABAergic neurons and enrichment for axonogenesis- and synaptic-related gene ontology categories, whereas hypoDMGs were enriched for transcription factor activity- and gene expression regulation-related terms. Finally, we found that the DNAm-based “epigenetic age” of neurons from heroin users was younger than that in controls. Suicide-related results were more difficult to interpret. Collectively, these findings suggest that the observed DNAm differences could represent functionally significant marks of heroin-associated plasticity in the OFC.

## 1. Introduction

Opioid overdose is now the second leading cause of accidental death among adults in the U.S. [1,2]. Prescription opioids are believed to have served as a gateway to the use of heroin which is more readily available and less expensive than prescription medications [3,4,5]. The opioid epidemic has raised attention to the relatively limited knowledge about the pathophysiology underlying heroin use disorder, particularly from insights gained through interrogation of the human brain.

As with other drugs of abuse, the susceptibility to opioid addiction is known to be influenced roughly equally by genetic and environmental factors [6,7,8], suggesting an important role for epigenetic regulation. Epigenetic mechanisms mediate long-term changes in gene expression without changes in DNA sequence [9,10]. Recent studies in animal models provide robust evidence that repeated exposure to drugs of abuse induces changes in gene expression through alterations in all major modes of epigenetic regulation (e.g., cytosine DNA methylation (DNAm), histone modifications, and non-coding RNAs), and in several instances, the contribution of such epigenetic changes to addiction-related behavioral abnormalities in animals has been directly demonstrated [11]. Specific examples include changes in the level of the transcription-activating histone modification H3K27ac in different brain regions in several experimental models of drug addiction [12,13], alterations of the repressive histone marks H3K9me2/3 in the nucleus accumbens during chronic cocaine or opioid addiction [14,15], and a plethora of epigenetic mechanisms which have recently been implicated in the downregulation of BDNF expression in the ventral tegmental area after chronic opioid exposure [16]. 

DNAm is a stable mostly repressive epigenetic modification that is extremely important for both the establishment of cell-type-specific phenotypes in the nervous system [17] and the mediation of environmentally induced changes in the adult brain including memory formation, stress responses, and depression [18,19,20]. Changes in levels of DNAm at specific gene loci, as well as changes in levels of modifying proteins, have also been observed after exposure to drugs of abuse, with a recent study supporting the role of DNA hydroxymethylation (5 hmC) in chronic cocaine addiction [21,22]. Whereas such information from animal studies is valuable, increasing the limited knowledge about the DNAm landscape in the brains of humans with substance use disorders is critical for the development of novel more effective treatments. 

Several features of DNAm were considered in the design of the present study. First, recent studies indicate that the composition and dynamics of DNAm are not only distinct in the brain compared to other tissues [23,24], but also differ significantly across different regions of the brain [25,26]. Studies of cognitive processes that accompany drug addiction during the last decade suggest that addiction involves neuroplasticity mechanisms similar to traditional models of learning and memory [27] and that these mechanisms underlie the role of the medial (*m*) prefrontal cortex (PFC) in drug self-administration and the long-lasting propensity to relapse [28,29,30]. Moreover, human imaging studies [31,32,33] and rodent studies that employed a reinstatement model of drug relapse [34,35,36] strongly suggest involvement of the ventral aspects of the mPFC in addiction. In particular, these studies suggest that diminished output from the ventral mPFC contributes to drug seeking behavior by impairing the ability to actively inhibit behavioral responses to drug-conditioned stimuli. For these reasons, in the present study we examined DNAm in autopsy specimens from the medial orbital frontal cortex (mOFC)—a ventral subregion of the mPFC—which participates in regulating goal-directed behavior and decision-making and has been implicated in drug addiction by human imaging and animal studies [37,38].

In addition, a number of studies have reported DNAm alterations in the human brain that are associated with addiction (specifically with alcohol use disorder, e.g., [39,40]). These studies were performed using bulk (cellularly heterogeneous) tissues. However, several recent reports have clearly demonstrated robust differences in DNAm and histone modification patterns between neuronal and glial cell populations in human and rodent brains [24,41,42]. These cell type-specific epigenetic landscapes might ultimately determine the selective vulnerability to neurodevelopmental or environmental insults that could culminate in drug addiction. Thus, in order to increase the likelihood of identifying cell type-specific signatures of heroin addiction-associated epigenetic variations, our studies employed neuronal cells that were separated from glia using fluorescence activated nuclei sorting (FANS).

Recently, a highly accurate multi-tissue epigenetic biomarker of tissue age (“epigenetic clock”, also known as “Horvath clock”) based on DNAm levels has been introduced [43]. This approach allows one to estimate the “epigenetic age” of any organ, tissue, or cell type including sorted neurons. Mathematically it is defined as a weighted average across 353 CpG sites. The resulting age estimate (in units of years) is referred to as “DNA methylation age” (DNAm age) or “epigenetic age”. Recent studies support the idea that epigenetic age estimates can serve as biomarkers of biological age. For instance, the epigenetic age of blood has been found to be predictive of all-cause mortality [44,45,46,47,48], frailty [49], lung cancer [50], and cognitive and physical functioning [51]. Further, the utility of the epigenetic clock method using various tissues and organs has been demonstrated in applications surrounding Alzheimer’s disease [52], centenarian status [47,53], development [54], Down syndrome [55], HIV infection [56], Huntington’s disease [57], obesity [58], lifetime stress [59], and Parkinson’s disease [60]. It was also shown that different brain regions have different DNAm age, with the cerebellum displaying the youngest age of all tested regions [53].

In the present report, we compared DNAm in neuronal populations of the mOFC among individuals who abused heroin and died of heroin overdose, suicide completers without any evidence of heroin use, and a control group consisting of specimens from psychiatrically normal individuals who died of non-suicide causes and did not abuse illicit drugs. All brain specimens came from the same brain collection. Suicide completers were included as a comparison group with a different pathophysiology. The study revealed significant methylation disturbances in heroin abusers that may represent a functionally relevant epigenetic signature of heroin addiction in the human brain. Moreover, while the epigenetic age of neurons from the suicide and control individuals did not differ from their biological age, the epigenetic age of neurons from heroin users was younger. 

## 2. Materials and Methods 

### 2.1. Subjects and Tissue

Human postmortem brain specimens were obtained from our Brain Collection at the Icahn School of Medicine at Mount Sinai that has been extensively used for many molecular and epigenetic studies [42,61,62,63,64,65,66,67,68,69]. Brains were collected at autopsy at the Department of Forensic Medicine, Semmelweis University (Hungary) or National Institute of Forensic Medicine, Karolinska Institutet (Sweden). All material was obtained under approved local ethical guidelines. The cohort (*N* = 88) consists of a Hungarian/Swedish population of heroin abusers who died of heroin overdose (*N* = 37), suicide completers without any evidence of heroin use (*N* = 22), and control subjects who did not abuse illicit drugs and died of non-suicide causes (such as cardiac failure, viral infection, or an accident; *N* = 29) (Supplementary Materials Table S1). One of the control subjects was later excluded from the analyses due to ambiguity of sex, which was discovered during the data processing. The cause and manner of death and possible psychiatric diagnoses were determined by a forensic pathologist after evaluating autopsy results, circumstances of death, data from extensive toxicological testing, police reports, family interviews, and medical records. Exclusion criteria were postmortem interval (PMI) of >24 h, HIV-positive status, history of alcoholism, use of illicit drugs (other than heroin in heroin abusers) and the presence of Axis 1 psychiatric disorders. All specimens from heroin users included in the study were from individuals who died from heroin intoxication. These individuals were not receiving methadone or buprenorphine treatment, and had positive blood and/or urine levels for opiates at the time of death. Their average time of heroin use where this information was available (*N* = 23 individuals) was 3.75 years (ranging from 0.5 to 10 years). The control and suicide groups were negative for blood opiates and had no history of any drug addiction. All suicide subjects died from hanging. The control subjects died from myocardial infarction, pulmonary embolism, electric shock or viral infection. Subjects from all 3 groups showed negative toxicology for other common drugs of abuse and common therapeutic agents. EtOH was detected in the blood and/or urine of 6 heroin, 5 suicide, and 3 control subjects and EtOH levels were not significantly different among groups. Nicotine toxicology was not conducted, but tobacco use is frequent in the general population from which all the subjects were collected. 

Autopsy brain specimens were cut coronally in 1 cm slabs, frozen, and kept at −80 °C. From these specimens we harvested the ventral extent of the PFC commonly referred to as the orbital frontal cortex (OFC; Brodmann area 11). Specifically, we dissected the regions containing the medial orbital (MOrG) and inferior orbital (IOrG) gyri just anterior to the transversely running orbital sulcus. The region was dissected in a single block bounded by the olfactory sulcus medially and the inferior orbital sulcus laterally as described previously [42].

### 2.2. Nuclei Separation by Fluorescence Activated Nuclei Sorting (FANS) 

Cell structure is not preserved in frozen autopsy brain specimens. However, the nuclei of different cell types remain intact. Antibodies against the RNA-binding protein NeuN, which is expressed exclusively in the neuronal nuclei, have been used to separate neuronal from glial nuclei using FANS (e.g., [24,41,70,71,72,73]). In a recent study, we optimized previously published FANS protocols by employing the DNA-binding dye 7-AAD and anti-NeuN antibodies directly conjugated with the fluorophore [42]. This protocol was used in the present study. In short, for each specimen, mOFC tissue was ground using mortar and pestle on liquid nitrogen, resuspended in ice-cold Lysis Buffer (0.1% Triton, 0.32 M sucrose, 5 mM CaCl_2_, 3 mM MgCl_2_, 10 mM Tris-HCl), filtered through a cell strainer, and centrifuged for 5 min at 300 g. The pellet was resuspended in Blocking Buffer (1% goat serum, 2 mM MgCl_2_, TBS) and incubated for 45 min with Alexa488-conjugated anti-NeuN antibodies (EMD Millipore, Billerica, MA, USA) (1:1000 dilution). Next, a second centrifugation step (15 min, 2800 g) through a layer of 1.1 M sucrose was done, and the resulting pellet was resuspended in PBS. The DNA dye 7-AAD (Sigma-Aldrich, St. Louis, MO, USA) was added to a final concentration of 2 μg/mL, and the sample was subjected to the FANS procedure using Vantage with DiVa (excitation wavelength 488 nm). Finally, the sorted nuclear fractions were precipitated by centrifugation at 4000 rpm for 20 min at 4 °C and stored frozen at −80 °C until DNA isolation. The latter was performed using proteinase K treatment and two rounds of phenol extraction followed by ethanol precipitation. 

This protocol allowed us to routinely obtain well-separated NeuN(+) and NeuN(−) nuclear fractions, with the width of separation reaching up to an order of magnitude of the NeuN signal intensity [42]. In addition, the DNA content of both fractions was well-defined because aggregates, nuclei of dividing cells, and debris were excluded in the process of sorting. Validation of the cell-type specificity of the obtained neuronal and non-neuronal populations was done previously by demonstrating the enrichment for the known neuronal or glial-specific transcripts in RNA samples extracted from the sorted NeuN(+) and NeuN(−) populations, respectively [42]. We estimated the proportions of neurons and glia in our FANS-separated neuronal nuclear preparations using the algorithm from [74] and NeuN(+) and NeuN(−) reference data from [41] (see [75] for details). For comparison, we also included DNA methylation data for 6 NeuN(−) (glial) specimens from our published study [42]. The results are presented in a Supplementary Materials Figure S1, and demonstrate high purity of our neuronal nuclear preparations.

### 2.3. DNA Methylation Measurement and Analysis 

For each specimen, DNA was extracted from the neuronal fraction and subjected to sodium bisulfite treatment to generate methylation-specific base changes before hybridization. Batch effect was minimized by performing the bisulfite treatment simultaneously for all 88 specimens, and by randomizing the placement of heroin, suicide and control samples across the arrays.

DNA samples were bisulfite converted using EZ DNA methylation kit (Zymo Research, Tustin, CA, USA). Specifically, 500 ng of high quality genomic DNA (A260/260 ≥ 1.8; A260/230 = 2.0–2.2) was denatured by incubation with NaOH-containing Zymo M-Dilution buffer for 15 min at 37 °C. Next, the denatured DNA was incubated with bisulfite-containing CT-conversion reagent for 16 h at 50 °C in a thermocycler. Every 60 min the reaction was heated to 95 °C for 30 s. All 88 samples were processed on the same plate. The Infinium methylation assay was carried out as described [76]. In short, 4 μL of bisulfite-converted DNA (~150 ng) was used in the whole-genome amplification reaction. After amplification, the DNA was fragmented enzymatically, precipitated and re-suspended in hybridization buffer. All subsequent steps were performed following the standard Infinium protocol (User Guide part #15019519 A). The fragmented DNA was dispensed onto the HM450K Bead-Chip [77], which was followed by hybridization in a hybridization oven for 20 h. After hybridization, the array was processed through a primer extension and an immunohistochemistry staining protocol to allow detection of a single-base extension reaction. Finally, the Bead-Chip was coated and then imaged on an Illumina iScan (Illumina, San Diego, CA, USA). The same iScan array scanner was used for processing all samples. 

All 88 samples that were used in this study passed Illumina quality control requirements and received a status of “Successful Sample”. The data were further evaluated for quality using the “minfi” R/Bioconductor package [78]. All samples passed by the suggested criteria of median *M* and *U* intensities greater than 10.5. All but one sample passed a test for concordance between estimated and reported sex. This sample was removed from the subsequent analyses (see Supplementary Materials Table S1). The data were then pre-processed with stratified quantile normalization described in the minfi paper [78]. Probes with annotated/dbSNP-labeled SNPs in the single base extension or target CpG site were filtered, as were probes on the sex chromosomes, leaving 456,513 probes for subsequent analysis. 

For differential methylation analysis, we employed limma R/Bioconductor package to perform linear regression and moderated t-statistics (with empirical Bayes) adjusting for age, sex and tissue pH and the first four “negative control” principal components, which typically capture batch and slide effects as described previously [75]. Differentially methylated (DM) CpG sites (DMSs) were assigned to genes using the *distanceToNearest* function in the GenomicRanges R package (http://bioconductor.org/packages/release/bioc/html/GenomicRanges.html) and the KnownGene data set from the University of California, Santa Cruiz) (UCSC) (http://genome.ucsc.edu).

### 2.4. Estimation of DNA Methylation Age

DNAm age (also referred to as epigenetic age) was calculated from the neuronal samples profiled with the Illumina Infinium 450 K platform as described in [43]. Briefly, the epigenetic clock is defined as a multivariate linear model for predicting age based on the DNAm levels of 353 CpGs. These CpGs and their weights (coefficient values) were chosen using several independent data sets by regressing chronological age on CpGs. Predicted age, referred to as DNAm age, correlates with chronological age in sorted cell types (CD4+ T cells, monocytes, B cells, glial cells, neurons), tissues, and organs, including: whole blood, brain, breast, kidney, liver, lung, and saliva [43]. In our study, the epigenetic clock method was implemented using publicly available *R* software scripts and in a web-based calculator. The measure of epigenetic age acceleration was defined as a raw residual resulting from regressing DNAm age on chronological age. By definition, epigenetic age acceleration does not correlat with chronological age (*r* = 0).

### 2.5. Gene Ontology Functional Annotation Analysis

Gene ontology (GO) analysis was performed with the online software tool WebGestalt (www.webgestalt.org, [79]). Enriched GO terms with adjusted *p*-values < 0.01 (Benjamini-Hochberg multiple test adjustment) were considered statistically significant. In addition, we applied the cutoff of *N* ≥ 10 genes for the minimal number of genes associated with a specific GO term. For the GO analysis, we included the genes which had both hyperDMSs and hypoDMSs into both hyperDM gene (G) and hypoDMG lists. 

### 2.6. Gene Expression Comparison between Glu- and GABA Neurons in the Human PFC

We employed our recently developed FANS-based protocol [69] to isolate two types of neuronal nuclei: (1) the MGE-derived GABA neurons, and (2) glutamatergic neurons with a small (~10%) admixture of non-MGE derived GABA neurons (denoted “Glu neurons”). PFC tissue samples from 3 control subjects were used for the experiments. After nuclear isolation, we employed an optimized version of our RNA isolation protocol (see [69]). Specifically, 40,000 nuclei were sorted directly into 150 uL of Extraction Buffer from the PicoPure RNA isolation kit (ThermoFisher Scientific, Waltham, MA USA). RNA isolation was then performed according to the kit protocol, with the inclusion of the on-column DNAse treatment step, and the RNA was eluted in 15 uL Elution Buffer. RNA-seq libraries were then prepared from 10 ng RNA using the SMARTer Stranded Pico RNA-seq library preparation kit (Clontech, Mountain View, CA, USA), and sequenced on a HiSeq 2500 Illumina sequencer (Illumina, San Diego, CA, USA) using paired-end 50 bp protocol. FASTQ files were trimmed to remove low quality reads and adapters (using Scythe and Sickle software packages (https://github.com/vsbuffalo/scythe, https://github.com/najoshi/sickle)), further trimmed by 3 bp from the 5′ end of read 1 as suggested in the library kit protocol, and then mapped to human hg19 genome with STAR software [80]. EdgeR R package [81] was used to perform differential expression (DE) analysis between GABA and Glu neurons, with DE criteria of FDR < 0.01, abs(FoldChange) > 2, and a cutoff of 0.1 counts per million (CPM). Supplementary Materials Table S2 presents the resulting lists of the DE genes.

## 3. Results

### 3.1. mOFC Neurons of Heroin Users Show Younger Epigenetic Age Than Neurons of Non-Addicted Individuals

**Figure 1:**
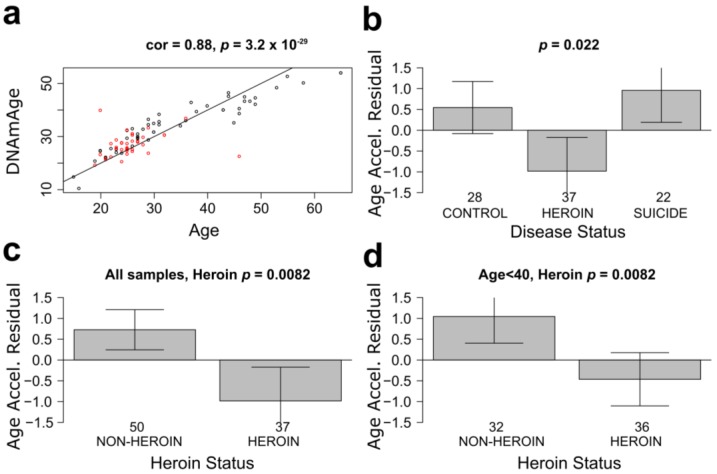
Neurons of heroin users show younger epigenetic age than neurons of non-addicted individuals. (**a**) Correlation of neuronal DNAm age with chronological age of all subjects at time of death. Red circles, heroin subjects; black circles, all other subjects; (**b**) Analysis of epigenetic age acceleration in the neurons among heroin users, suicide subjects, and controls. The heroin subjects have a younger DNAm vs. chronological age (*p* = 0.022); (**c**) Analysis of epigenetic age acceleration in the neurons comparing heroin users and all subjects who did not abuse heroin. A significantly younger neuronal DNAm age in heroin users was observed (*p* = 0.0082); (**d**) Same as in (**c**), but for subjects with chronological age <40. The titles of the bar plots report the results of a non-parametric group comparison test (Kruskal Wallis test).

It has been suggested that epigenetic age acceleration (that measures deviations between DNAm age and chronological age) captures aspects of the biological age of the brain tissue [43]. We estimated the epigenetic age (also known as DNAm age) of each neuronal mOFC sample by averaging the DNAm levels of 353 CpGs profiled with the Illumina Infinium 450 K assay. As expected, neuronal DNAm age was highly correlated with chronological age of subjects at the time of death across all samples (correlation *r* = 0.85, *p* = 1.2 × 10^−25^; Figure 1a). We defined a measure of epigenetic age acceleration as residual resulting from regressing DNAm age on chronological age. Thus, a positive value of age acceleration indicates that the DNAm age of a sample is higher than expected based on chronological age, whereas a negative value indicates that the DNAm age is younger than expected based on chronological age. Whereas there were no differences in the neuronal age acceleration between the control (*N* = 28) and suicide subjects (*N* = 22), the heroin subjects (N=37) showed a younger DNAm vs. chronological age (*p* = 0.022; Figure 1b). A significantly younger neuronal epigenetic age in heroin abusers was observed when we compared their age acceleration to that of all subjects who did not abuse heroin (controls and suicides, *N* = 50; *p* = 0.0082; Figure 1c). The difference in age acceleration was also observed when only young adults (subjects with chronological age <40) were considered in the analysis (*N* = 32 and *N* = 36 for non-abusers and abusers, respectively; *p* = 0.0082; Figure 1d). Because in our cohort the average age of heroin individuals is younger than control individuals, the latter analysis provided additional validation of our findings using groups that were better balanced by age.

### 3.2. Differential DNA Methylation Analysis

We compared samples from heroin users, suicide completers and control individuals to identify differences in DNAm, adjusting for age, sex and tissue pH as covariates. After correcting for multiple comparisons, we did not identify any differentially methylated (DM) CpGs sites between control and suicide specimens using FDR < 0.1, whereas there were 3 sites that were DM between heroin users and controls. We then used a more liberal threshold of significance (nominal *p*-value < 0.001). This resulted in 454 DM sites (DMSs) between suicide and control subjects (DMsui sites) and 1311 sites that were DM between heroin users and controls (DMher sites) (Supplementary Materials Table S3), and we used these DM sites to focus on gene-set-level analyses. Among the DMher sites, there were 12 DMSs that belonged to the CpH sequence context; these were excluded from the subsequent analysis, retaining 1298 DMher CpG sites. The absolute differences in average DNAm levels (beta values) were generally small. Nevertheless, the majority of DMSs (1247 out of 1298 for DMher, and 431 out of 454 for DMsui sites) showed beta value differences >1%, and the smallest beta value difference among the remaining DMSs was 0.62%. Because of the relatively small number of DMSs with low (<1%) differences, we retained all DMSs for the subsequent analyses. The DMher and DMsui sites represented largely non-overlapping populations, with only 29 CpG probes showing significant DNAm changes in both heroin users and suicide subjects compared with controls (Supplementary Materials Table S4). 

In our cohort, the average age of heroin individuals was younger than control individuals. In addition to including age as a covariate in our initial analysis, we also performed a secondary analysis in order to better account for the role of age on our results. Specifically, we left out *N* = 10 oldest control individuals to balance the groups by age, analyzing *N* = 18 controls and *N* = 37 heroin individuals. In this new cohort there were no significant difference in age between the heroin and control subjects (*p* = 0.09). We detected no noticeable effect of age on the regression estimates for the heroin vs. control comparison (Supplementary Materials Figure S2), suggesting that we had properly adjusted for age effects in the initial differential methylation analysis.

The information about the years of heroin use was available only for a subset of heroin subjects. In order to test if the duration of heroin abuse influenced the differential methylation detected in our study, we left out the heroin individuals (*N* = 14) which did not have the years-of-use information, and using this filtered cohort, compared the results of the differential methylation analyses employing two different linear models: (1) heroin/non-heroin status, and the covariates from the initial analysis, and (2) Log10 (Years of Heroin Use + 1) and the covariates from the initial analysis. We found no significant differences between the results from these two models (Supplementary Materials Figure S3).

We then defined differentially methylated genes (DMGs) based on the position of the DMSs (see Materials and Methods). There were a total of 1159 unique DMGs associated with DMher sites (DMher genes) and 407 genes associated with DMsui sites (DM genes) (Supplementary Materials Table S5); 87 genes were common for both DMher and DMsui gene lists. Among the DMher genes, 660 were associated with at least one hyperDMS and 531 were associated with at least one hypoDMS, with 32 DMher genes associated with both a hyper- and a hypoDMS. There were 243 hyperDMsui genes and 170 hypoDMsui genes, with 6 DMsui genes overlapping between these two lists (Supplementary Materials Table S5). The DMGs that were present in both the hyperDMG and hypoDMG lists were excluded from the analysis for neuron subtype specificity (see below).

### 3.3. Enrichment of Differentially Methylated Sites in Genomic Features and Regulatory Elements

We first asked if heroin- or suicide-associated hyper- or hypoDMSs were enriched (or depleted) within genic features (i.e., promoters, exons, introns, entire gene bodies, intergenic regions), putative regulatory elements (i.e., predicted enhancers) or CpG island-related features (i.e., CpG islands, shores, or regions outside of islands and shores). We also tested if such enrichments could be specific to the direction of the DNAm changes (hyper- vs. hypoDM).

We detected clear differences in the patterns of enrichment or depletion between hyper- and hypoDMSs within many of the assessed features in the neurons from heroin users (Table 1, statistical significance by Fisher’s exact test). In particular, within genic features, hyperDMher sites were strongly enriched in exons and gene bodies, moderately enriched in introns, but significantly depleted from promoters. In contrast, hypoDMher sites were enriched in promoters, but did not show significant enrichment or depletion in other genic features.

Likewise, we detected differences between hyper- and hypoDMher sites in their pattern of enrichment or depletion within putative transcriptional enhancers. The genomic coordinates of these enhancers were based on the ChIP-seq peaks for the H3K27ac histone mark in the human PFC tissue (data obtained from the NIH Roadmap Epigenomics Mapping Consortium, REMC [82]). We performed this analysis separately for the H3K27ac peaks distal from transcription start site (TSS) (>1000 bp distance), which are indicative of putative active enhancers, and for the TSS-proximal H3K27ac peaks, which are indicative of active promoters. The hypoDMher sites were strongly enriched within distal peaks (putative enhancers), whereas the hyperDMher sites showed neither enrichment nor depletion in putative enhancers. In line with their enrichment in promoters, the hypoDMher sites were enriched in proximal H3K27ac peaks, whereas the hyperDMher sites were strongly depleted from both promoters and proximal H3K27ac peaks.

Both hyper- and hypoDMher sites were depleted from the genomic areas outside of CpG islands or shores (the latter is defined as 2000 bp-width-areas at ≤2000 bp from the nearest CpG island). In addition, hyperDMSs were slightly enriched in CpG islands, whereas hypoDMSs were enriched within shores.

When the distribution of the suicide-associated DMSs within different genic features was analyzed, only few significant results were detected. HyperDMsui sites were depleted within promoters as well as within proximal H3K27ac peaks, and were moderately enriched in exons, introns and gene bodies (Table 1). No enrichment or depletion within any genic feature was found for hypoDMsui sites, which might be explained by the relatively small number of DMs within this group.

### 3.4. Gene Ontology Analysis of Differentially Methylated Genes

To obtain insight into the possible biological implications of the detected DNAm changes in the OFC of heroin users and suicide completers, we performed a functional annotation and enrichment analysis of the DMGs using the online software tool WebGestalt (www.webgestalt.org [79]). This tool integrates information from a number of public resources to assess the enrichment of gene sets in various functional categories, and presents the results as a list as well as a tree-like diagram displaying the hierarchical relationship of the enriched categories. We focused our analysis on the gene ontology (GO) module of WebGestalt, which comprises 3 large subsets of GO terms: “Biological Process”, “Molecular Function” and “Cellular Component”.

Notably, when hyperDMher genes (*N* = 660) and hypoDMher genes (*N* = 531) were analyzed, we obtained essentially non-overlapping sets of enriched GO terms (Supplementary Materials Table S6 and Figure S4A,B). Among the “Cellular_Component” GO subclass, the GO localization terms associated with axons or synaptic compartments (“axon”, “synaptic membrane”) were enriched with the hyperDM but not with the hypoDM gene set. In contrast, several GO categories associated with gene expression regulation and transcription factor activity (“transcription factor binding”, “sequence-specific DNA binding RNA polymerase II transcription factor activity” and others) were enriched in the hypoDM but not in the hyperDM gene sets. In addition, “Biological_Process” GO terms “transmission of nerve impulse”, “axonogenesis” and “cell–cell signaling” were specific to the hyperDM genes. On the other hand, the hypoDM genes were enriched for the “regulation of neuron differentiation” GO term, which included several transcription factors and growth factors (e.g., *NGF*).

When suicide-associated DMGs were analyzed, no significant enrichments were found for the hypoDM gene set, which was only comprised of 170 genes. The hyperDMsui genes were enriched for the “synaptic transmission” GO term, as well as for several other broad GO terms not directly related to the nervous system function (Supplementary Materials Figure S4C).

### 3.5. Neuronal Subtype-Specificity of Hyper- or Hypomethylated Genes 

Our analysis of DM between heroin users or suicide subjects and controls was performed in FANS-separated neuronal nuclei. This experimental design could not distinguish between the contributions of specific neuronal subpopulations that exist in human PFC (e.g., excitatory glutamatergic or inhibitory GABAergic neurons) to the observed differences in DNAm. To investigate neuronal subtype-specificity of the hyper- and hypoDM genes, we compared the DMG sets with genes that were differentially expressed (DE) between Glu projection neurons and MGE-derived GABA interneurons in the PFC. MGE-derived GABA interneurons comprise ~60–70% of all cortical GABA neurons, and express parvalbumin (*PVALB*) or somatostatin (*SST*) [83]. This population of GABA neurons constitutes an essential component of inhibitory neuronal networks in the human and mammalian cortex and has been implicated in many neurological and psychiatric diseases (including schizophrenia, major depression, autism, and epilepsy) [84,85,86,87]. Therefore, it was important for the interpretation of the results of our study to classify the DMGs as GABA- or Glu-specific. To this end, we employed the FANS-based nuclei isolation protocol that was recently developed in our lab [69] and that allowed us to separate and obtain neuronal nuclei from these neuronal populations. PFC tissues obtained from 3 control subjects were analyzed.

The DE analysis identified a number of genes that were mostly expressed in Glu or GABA neurons (Glu-specific and GABA-specific DE genes (DEGs), *N* = 1339 and 817 genes, respectively), Next, we overlapped the DEG and DM gene sets and assessed the enrichment using the hypergeometric distribution test. A significant difference in patterns of enrichment was observed between hyper- and hypoDM genes (Table 2). For both DMher and DMsui genes, the hyperDMGs were strongly enriched for Glu-specific DE genes, but not for GABA-specific genes (Table 2). In contrast, hypoDMher and hypoDMsui genes showed no enrichment for Glu-specific DEGs. We, however, detected that hypoDMsui genes were significantly enriched for GABA-specific DEGs (Table 2).

## 4. Discussion

In this study we employed the Illumina 450 K Infinium DNA methylation array to probe the differences in DNAm in the OFC of heroin abusers who died of heroin overdose, suicide completers, and control subjects who died of non-suicide causes. There was no evidence of any illicit drug use for the latter two groups. We analyzed differential DNAm (1) between suicide completers and controls and (2) between heroin users and controls. Applying a conservative nominal *p*-value (<0.001) we detected 454 CpG sites that were DM between suicide and control subjects, and 1298 CpG DM sites between heroin users and controls. We then assessed genomic features, neuron-subtype specificity as well as functional categories and pathways that were associated with these DNAm differences.

Whereas the number of DM sites and genes identified in the suicide vs. control comparison was low and the results were difficult to interpret, we found noticeable differences for heroin-associated DM sites and genes. In particular: (1) hyperDMher sites preferentially occurred in exons and more generally in gene body regions. In contrast, no such enrichments were detected for hypoDMher sites; (2) hypoDMher sites were significantly enriched in promoters and in the TSS-proximal H3K27ac ChIP-seq peaks, whereas hyperDMher sites were dramatically depleted in these regions; (3) hypoDMher sites were strongly enriched in TSS-distal H3K27ac ChIP-seq peaks (predicted enhancers), whereas hyperDMher sites were not significantly overrepresented in these regulatory regions; (4) at the gene level, hyperDMher genes were strongly enriched in Glu-specific, but not in GABA-specific genes; (5) significant differences in gene ontology (GO) category enrichments were found between hyper- and hypoDMher gene sets: hyperDMher genes were enriched in GO categories for genes implicated in axonal and synaptic localization, as well as in synaptic transmission. In contrast, hypoDMher genes were not enriched in these categories. Instead, hypoDMher sites were overrepresented among genes with DNA binding and transcription factor activity. Thus, the hyper- and hypomethylation in the specimens from heroin users are enriched in different neuronal subtypes and different genomic contexts. These observations imply that different mechanisms of epigenetic regulation might be recruited in different neuronal subtypes or specifically targeted to particular genic features (promoters, exons, enhancers, etc.) in the context of opioid exposure. Collectively, these findings suggest that the observed DNAm differences could represent functionally significant marks of heroin-associated maladaptive plasticity in the OFC.

Notably, heroin-associated enrichment of the hyperDMGs in Glu-specific genes as well as in synaptic and axonal genes is in agreement with the notion that drug-associated alterations in the ventral PFC lead to reduced glutamatergic output from this region [30]. Our findings are also in line with the recent study showing morphine-induced generation of silent (AMPA-receptor-deficient) excitatory synapses in the rodent nucleus accumbens (NAc) [88]. These silent synapses were specifically generated from the existing matured synapses in the NAc D2-type medium spiny neurons (MSNs), and likely represented an intermediate step in the subsequent synaptic elimination, thus reducing the excitatory drive to the D2-type MSNs in the NAc from other brain regions. It is, therefore, plausible that our finding of increased DNAm at the genes involved in axonogenesis, axonal and synaptic function in the OFC of heroin users may represent a presynaptic mechanism contributing to the weakening and elimination of the excitatory synapses of the OFC projections to the NAc. It is important to note that the mechanism of the silent synapse formation described above was specific to morphine but was different in the case of cocaine treatment [88], and the cohort of the drug abusers employed in our study consisted exclusively of heroin users, who did not abuse other illicit drugs (e.g., cocaine or amphetamine).

Whereas our approach was mostly focused on the analysis of sets of DMSs or DMGs, inspecting these data allowed us to identify a number of individual genes that could potentially have major implications for heroin addiction or suicide. Among the heroin-associated DMGs are *SLC17A7* (gene coding for glutamate transporter; probe ID cg16890796), *OPRL1* (that encodes an opioid receptor-like protein, nociceptin receptor, which has been implicated in alcohol and other drug reward pathways [89]; cg24210478, cg23205874), as well as *TET3* (cg11236515). The latter encodes one of the 3 Ten-Eleven Translocation enzymes that oxidize methylcytosine to produce the hydromethylcytosine DNA mark, and thus could alter the inhibitory character of DNAm and/or contribute to DNA demethylation. Of note, *TET1*, another member of the TET family, has been recently implicated in the mechanism of cocaine action in the NAc [22]. Another heroin use-associated DMG is *ARC* (cg08387463). *ARC* is an immediate early gene (IEG), which can be upregulated in an activity-dependent manner [90]. It encodes Arc/Arg3.1 protein implicated in synaptic plasticity, and in particular, in the mechanisms of morphine addiction in the NAc [91]. Here we detected significant hypermethylation of a CpG site within the first exon of *ARC*, at ~1300 bp distance from the TSS. Increased DNAm of *ARC* (which contrasts with increased *ARC* expression in the NAc as found in other studies) might be a specific signature of heroin addiction in the OFC.

Among the suicide-associated DNAm genes, of particular interest are *PRRT1* and *SORBS2*. For both of these genes, more than one hyperDMS was detected in suicide completers vs. controls, (7 hyperDMSs for *PRRT1* (cg18419271, cg02925367, cg27067781, cg21035875, cg14757228, cg17626960, cg14531663)—the largest number of sites for any suicide-associated DMG in our study, and 3 hyperDMSs for *SORBS2*, cg09555153, cg04392082, cg22328746). *PRRT1* encodes a little-studied auxiliary subunit of the AMPA receptor, and was shown to colocalize with extrasynaptic GluA1 puncta in primary neuronal cultures [92]. The *SORBS2* gene encodes ArgBP2 protein and is alternatively spliced in the brain to encode a neuron-specific nArgBP2 isoform. In mice, nArgBP2 is highly expressed in many brain regions, including cortex, and localizes at dendritic spines. Knockout of *Sorbs2* leads to reduced dendritic complexity, as well as to a reduced acoustic startle response, and defective long-term object recognition memory and contextual fear memory [93]. It was also shown that nArgBP2 serves as a major regulator of dendritic spine morphology at excitatory synapses, potentially implicating this protein as a “hub” molecule in various neuropsychiatric disorders, including bipolar disorder [94].

Another intriguing finding of our study is the discovery that the OFC neurons of heroin users show a younger epigenetic age than the control subjects. This finding will require confirmation by other studies and is not straightforward to interpret. However, significant cellular and molecular changes were reported with repeated exposure to drugs of abuse, and the “neural rejuvenation” hypothesis has been recently proposed to conceptualize some of these changes, with one of the main defining features being the formation of immature, silent synapses [95]. Silent synapses can be generated de novo, as in the case of cocaine action in the NAc, but can also arise from existing, mature synapses, as was demonstrated in the case of morphine action (see above) [88,95]. It is plausible that the latter may be relevant to the differences in the neuronal epigenetic age between heroin abusers and controls uncovered in our study.

To the best of our knowledge, the present study constitutes the first genome-wide DNAm profiling of human postmortem brain samples (specifically, neuronal nuclei from the OFC) of heroin abusers vs. control subjects. It also contributes to a growing list of studies exploring DNAm in the suicide brain [96,97,98]. In future studies, it would be of much interest to explore some of the findings of the current work in independent (and larger) cohorts and using tissue specimens from different brain regions relevant for drug addiction. Such studies should preferably involve both epigenetic profiling and RNA-seq experiments, and, when possible, should be conducted using cell type- or subtype-enriched preparations.

## 5. Conclusions

We investigated the genome-wide DNAme differences in the OFC of individuals who abused heroin and died of heroin overdose and of individuals who did not use heroin and died by suicide vs. control subjects who did not use illicit drugs and died of non-suicide causes. We identified numerous CpG sites that were differentially methylated between heroin users and control individuals as well as between suicide victims and controls. We found that the hyper- and hypomethylated CpG sites and associated genes in the heroin users were enriched in different neuronal subtypes and different genomic contexts. These observations suggest that different modes of epigenetic regulation might be recruited in different neuronal subtypes or specifically targeted to particular genic features and/or regulatory elements (e.g., promoters, gene bodies or enhancers) in the context of heroin exposure. We also found that the DNAm-based “epigenetic age” of neurons from heroin users was younger than that in controls. Suicide-related differences in DNAm were subtle and difficult to interpret. Collectively, these findings implicate heroin-induced epigenetic remodeling in the previously reported functional abnormalities of the ventral mPFC in heroin abusers.

## Figures and Tables

**Table 1:** Enrichment or depletion of hyper- and hypomethylated sites (DMSs) within genomic features and putative enhancers. Statistical significance was calculated by Fisher’s exact test. Promoters were defined as regions within −1000 bp around the transcription start site. Significant (*p*-value < 0.01) enrichments or depletions are shown in italic bold. Moderately significant (*p*-value < 0.05) enrichments/depletions are shown in italic. H3K27ac ChIP-seq dataset was obtained from the Roadmap Epigenomics Mapping Consortium database (REMC, http://www.roadmapepigenomics.org).

Genomic Features/Putative Enhancers		Heroin DMSs				Suicide DMSs			
		HyperDMSs		HypoDMSs		HyperDMSs		HypoDMSs	
		Odds Ratio	*p*-Value	Odds Ratio	*p*-Value	Odds Ratio	*p*-Value	Odds Ratio	*p*-Value
Genic features	Exons	1.41	***1.6 × 10^−4^***	0.85	0.19	1.36	*0.045*	0.85	0.54
	Introns	1.21	*0.013*	0.97	0.75	1.31	*0.030*	1.15	0.38
	Gene Body	1.26	***0.0025***	1.06	0.52	1.29	*0.044*	0.94	0.70
	Intergenic	1.11	0.20	0.86	0.15	0.95	0.78	1.14	0.43
	Promoters	0.47	***4.8 × 10^−15^***	1.32	***0.0034***	0.53	***6.1 × 10^−5^***	0.83	0.34
H3K27ac ChIP-seq peaks (human PFC)	Distal H3K27ac peaks (enhancers)	1.18	0.07017	1.52	***3.0 × 10^−5^***	0.94	0.76	1.11	0.57
	Proximal H3K27ac peaks (promoters)	0.33	***2.0 × 10^−15^***	1.52	***1.2 × 10^−4^***	0.67	*0.048*	0.60	0.052
CpG island-related features	CpG islands	1.18	*0.03*	1.02	0.85	0.91	0.52	0.86	0.42
	Shores	1.16	0.075	1.22	*0.044*	0.86	0.42	0.85	0.29
	Other regions	0.76	***3.5 × 10^−4^***	0.85	0.06	1.22	0.12	1.19	0.26

**Table 2:** Overlap between heroin or suicide DMGs and Glu- or GABA-specific DEGs. The overlaps were calculated separately for hyper- and hypoDMGs. DMGs associated with both hyperDM- and hypoDM sites were not included. Statistical significance was calculated with the hypergeometric test. Significant overlaps (*p*-value < 0.01) are shown in bold. Only genes present in both the 450 K array annotation and the DE analysis data set were included in the analysis (13,544 genes).

DM/DE Genes		Glu-DE Genes	*N* = 1074 Genes	GABA-DE Genes	*N* = 701 Genes
		Overlapping	*p*-Value	Overlapping	*p*-Value
hyperDMGs	*N* = 537 genes				
heroin		70	**2.2 × 10^−5^**	27	0.59
hypoDMGs	*N* = 405 genes				
heroin		40	0.087	31	0.019
hyperDMGs	*N* = 201 genes				
suicide		34	**1.9 × 10^−5^**	11	0.47
hypoDMGs	*N* = 133 genes				
suicide		14	0.17	15	**3.6 × 10^−3^**

## Supplementary Materials

The following are available online at www.mdpi.com/2073-4425/8/6/152/s1, Table S1: Summary of demographics for the specimens used in the study, Table S2: Analysis of differential gene expression (DE) between Glu- and GABA-neurons, Table S3: Differentially methylated sites (DMSs) detected when comparing heroin users vs. control subjects, or suicide completers vs. control subjects, Table S4: Overlap between heroin associated- and suicide-associated DM sites (hyper- or hypoDMSs), Table S5: Differentially methylated genes (DMGs) between heroin users vs. control subjects, or between suicide completers vs. control subjects, Table S6: Gene ontology functional annotation analysis of DMG sets using WebGestalt tool, Figure S1: Estimated proportions of NeuN(+) and NeuN(−) nuclei in the FACS-separated neuronal nuclear preparations from the current study, Figure S2: Comparison of DM values for *N* = 1298 heroin vs. control DM sites between the initial analysis and the analysis which excluded 10 oldest control individuals, Figure S3: Assessment of influence of the duration of heroin use on the DM values for *N* = 1298 heroin vs. control DMSs, Figure S4: Graphic representation of the gene ontology annotation analysis of DMG sets using WebGestalt tool. (**A**): hyperDMheroin gene set, (**B**): hypoDMheroin gene set, (**C**): hyperDMsuicide gene set. No enriched gene ontology terms were found for hypoDMsuicide gene set. **Data Availability:** Data are available at GEO (GSE98203).
