# Supplementary material for: DNA Methylation Profiling of Human Prefrontal Cortex Neurons in Heroin Users Shows Significant Difference between Genomic Contexts of Hyper- and Hypomethylation and a Younger Epigenetic Age
Source: Genes (Basel). 2017 May 30;8(6):152. doi: 10.3390/genes8060152 (PMC5485516; doi:10.3390/genes8060152)
Supplement: Supplementary file 1 [file genes-08-00152-s001.zip › Figure S1.docx]

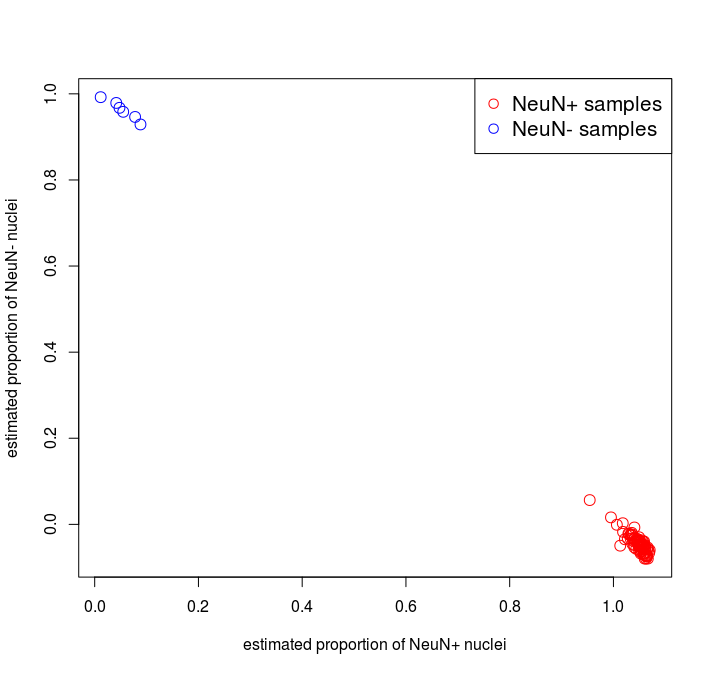


Suppl. File 1. Estimated proportions of NeuN(+) and NeuN(-) nuclei in the FACS-separated neuronal nuclear preparations from the current study. Algorithm from [1] and NeuN(+) and NeuN(-) reference data from [2] (see [3] for details) were employed. For comparison, HM450K DNA methylation data for 6 NeuN(-) (glial) specimens from [4] were included. Red circles—NeuN(+) FACS-separated preparations from the current study; blue circles—NeuN(-) preparations from [4]. The results demonstrate high purity of the neuronal nuclear preparations from the current study.

References:

[1] Houseman EA, Accomando WP, Koestler DC, et al. DNA methylation arrays as surrogate measures of cell mixture distribution. Bmc Bioinformatics. 2012;13:86.

[2] Guintivano J, Aryee MJ, Kaminsky ZA. A cell epigenotype specific model for the correction of brain cellular heterogeneity bias and its application to age, brain region and major depression. Epigenetics. 2013;8:290-302.

[3] Jaffe AE, Gao Y, Deep-Soboslay A, et al. Mapping DNA methylation across development, genotype and schizophrenia in the human frontal cortex. Nature neuroscience. 2016;19:40-7.

[4] Kozlenkov A, Roussos P, Timashpolsky A, et al. Differences in DNA methylation between human neuronal and glial cells are concentrated in enhancers and non-CpG sites. Nucleic Acids Res. 2014;42:109-27.
