# Supplementary material for: DNA Methylation Profiling of Human Prefrontal Cortex Neurons in Heroin Users Shows Significant Difference between Genomic Contexts of Hyper- and Hypomethylation and a Younger Epigenetic Age
Source: Genes (Basel). 2017 May 30;8(6):152. doi: 10.3390/genes8060152 (PMC5485516; doi:10.3390/genes8060152)
Supplement: Supplementary file 1 [file genes-08-00152-s001.zip › Figure S2.docx]

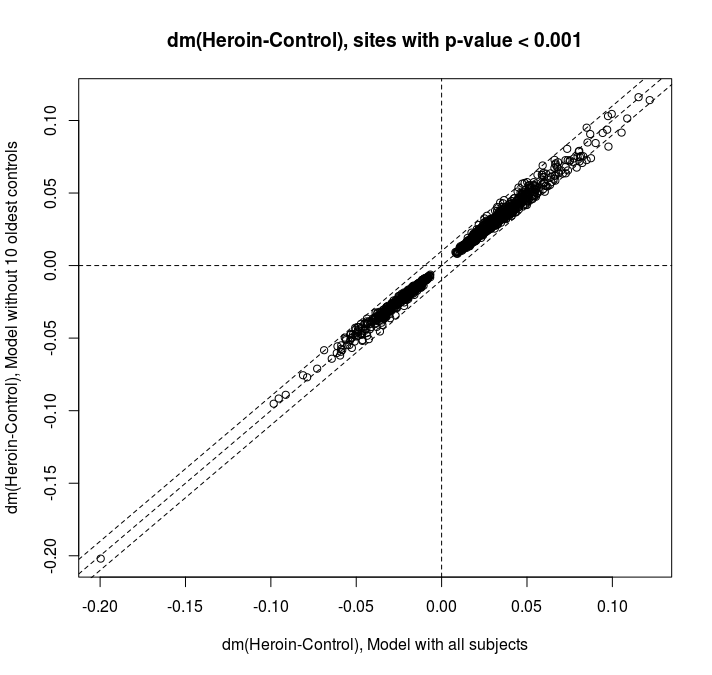


Suppl. File 2. Comparison of DM values for N=1298 heroin vs. control DM sites between the initial analysis (Cohort 1) and the analysis which excluded 10 oldest control individuals (Cohort 2). In Cohort 2, the average age of heroin (N=37) vs. control (N=18) subjects was not significantly different (p=0.09 by t-test). The two dotted lines parallel to the diagonal denote differences of 0.01 between the DM values in the two analyses: [delta(DM(Heroin – Control, Model 1) – DM(Heroin – Control, Model 2)].
