# Supplementary material for: DNA Methylation Profiling of Human Prefrontal Cortex Neurons in Heroin Users Shows Significant Difference between Genomic Contexts of Hyper- and Hypomethylation and a Younger Epigenetic Age
Source: Genes (Basel). 2017 May 30;8(6):152. doi: 10.3390/genes8060152 (PMC5485516; doi:10.3390/genes8060152)
Supplement: Supplementary file 1 [file genes-08-00152-s001.zip › Figure S3.docx]

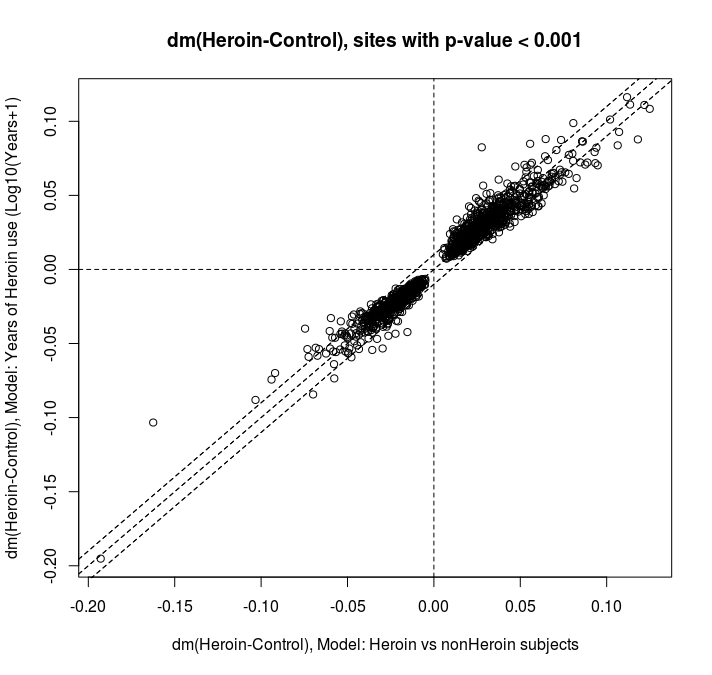


Suppl. File 3. Assessment of influence of the duration of heroin use on the DM values for N=1298 heroin vs. control DM sites. Heroin individuals (N=14) for whom the years-of-use information was not available were excluded from the analysis. Using this filtered cohort, two different linear models were employed for differential methylation analysis: ***(1)*** heroin/non-heroin status, and the covariates from the initial analysis, and ***(2)*** Log10(YEARS OF HEROIN USE+1) and the covariates from the initial analysis. No significant differences between the results from these two models were detected.
