# Supplementary figures and images for: DNA Methylation Profiling of Human Prefrontal Cortex Neurons in Heroin Users Shows Significant Difference between Genomic Contexts of Hyper- and Hypomethylation and a Younger Epigenetic Age
Source: Genes (Basel). 2017 May 30;8(6):152. doi: 10.3390/genes8060152 (PMC5485516; doi:10.3390/genes8060152)

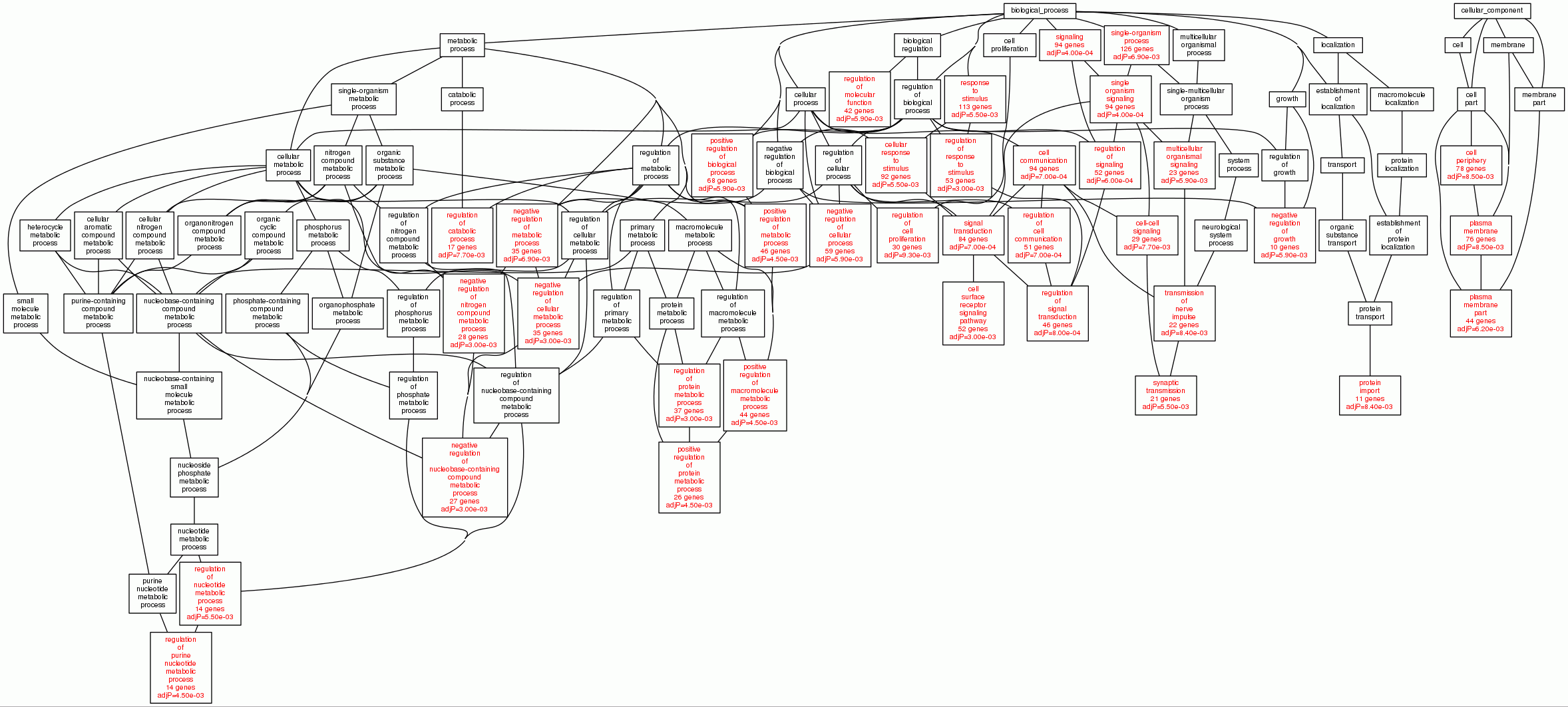

Supplement: Supplementary file 1 [file genes-08-00152-s001.zip › Figure S4C.gif]

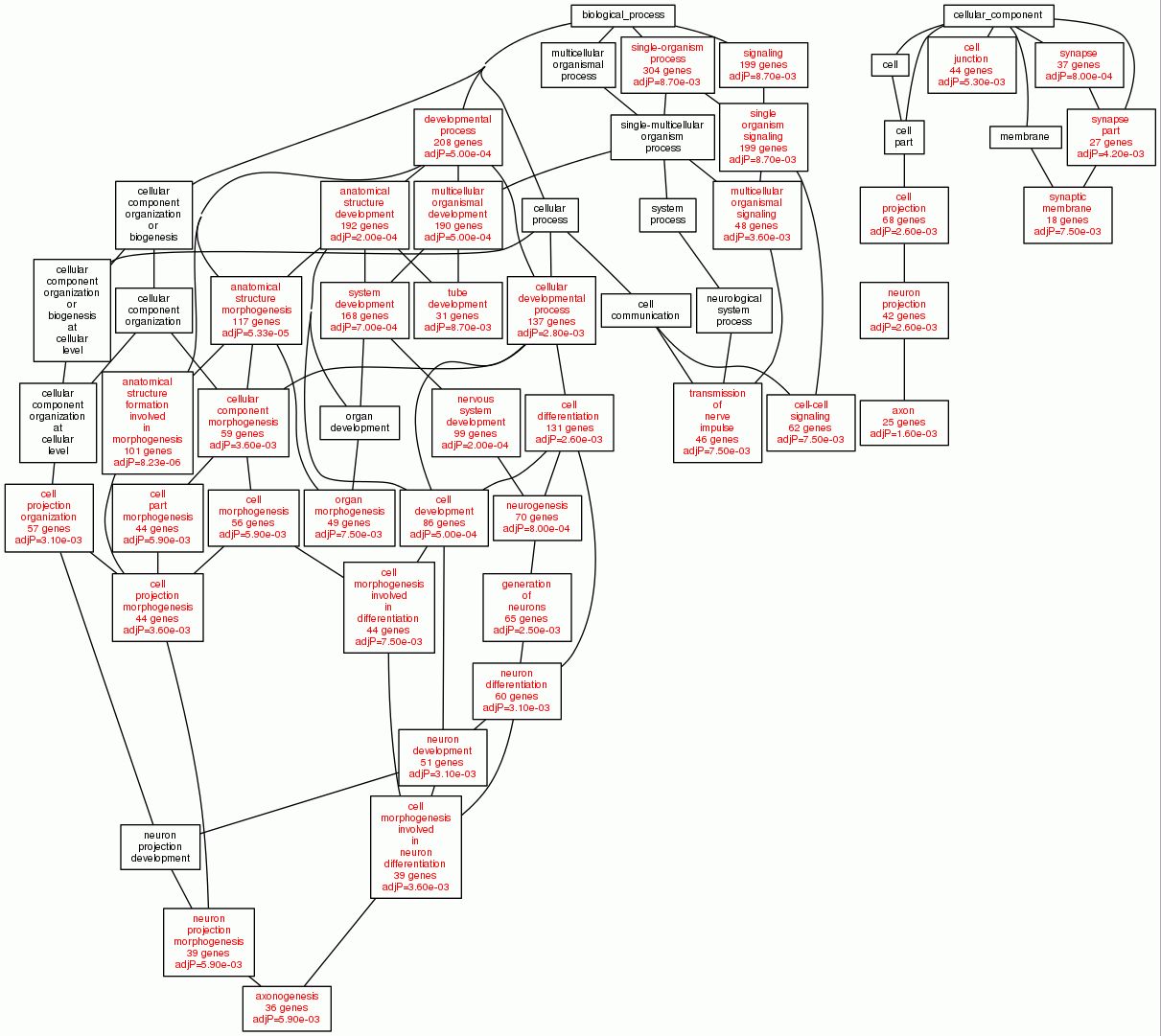

Supplement: Supplementary file 1 [file genes-08-00152-s001.zip › Figure S4A.gif]

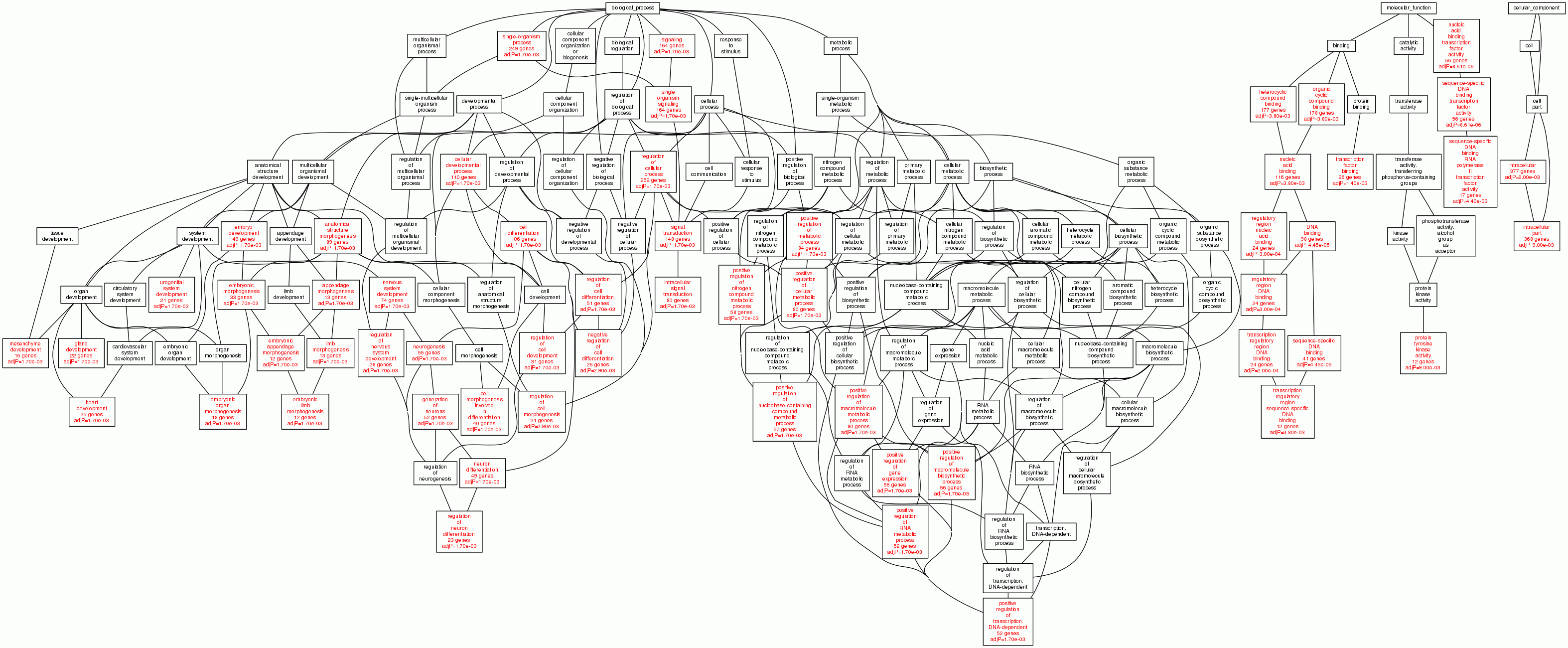

Supplement: Supplementary file 1 [file genes-08-00152-s001.zip › Figure S4B.gif]
